# Nonlinear regression modeling of ruminal fermentation dynamics in buffered diets using *in vitro* gas production

**DOI:** 10.1371/journal.pone.0336064

**Published:** 2026-06-18

**Authors:** Zahra Asadollahi Seyedabadi, Mohsen Danesh Mesgaran, Abbas Rohani, Seyed Alireza Vakili

**Affiliations:** 1 Department of Animal Science, Faculty of Agriculture, Ferdowsi University of Mashhad, Mashhad, Iran; 2 Department of Biosystems Engineering, Faculty of Agriculture, Ferdowsi University of Mashhad, Mashhad, Iran; Yantai Institute of Technology, CHINA

## Abstract

Understanding ruminal fermentation dynamics is essential for improving feed efficiency in ruminant nutrition. This study evaluated several nonlinear regression models to describe *in vitro* gas production kinetics in diets with different forage-to-concentrate ratios and buffer inclusion. Fermentation experiments were conducted using total mixed rations containing various concentrate levels, both with and without dietary buffers. Cumulative gas production data were fitted to exponential, logistic, Gompertz, and Michaelis–Menten models. Model performance was assessed using statistical goodness-of-fit criteria and the biological relevance of the estimated parameters. The results showed that buffer supplementation enhanced fermentation kinetics by stabilizing pH, increasing total gas production, and modifying volatile fatty acid profiles. Although no single model consistently outperformed others, model suitability depended on the specific diet–buffer combination. Sigmoidal models generally provided better fits for buffered diets, whereas exponential models were more accurate under high-concentrate, unbuffered conditions. These findings emphasize the importance of selecting context-specific models for analyzing ruminal fermentation data. The integration of *in vitro* gas production and nonlinear modeling offers a reliable approach to evaluate dietary strategies that improve nutrient utilization and feed efficiency in ruminant systems.

## Introduction

Nutrition is a key determinant of performance, health, and welfare in dairy cows [1]. To meet the high energetic demands of lactation and reproduction, high-producing cows are commonly fed energy-dense total mixed rations (TMRs) with high concentrate-to-forage ratios [2,3]. These diets enhance nutrient intake and milk yield [4], but they also induce substantial alterations in the ruminal environment. Such alterations include shifts in volatile fatty acid (VFA) profiles, suppression of cellulolytic microbial populations, and changes in biohydrogenation pathways. Together, these effects lower ruminal pH and markedly increase the risk of subacute ruminal acidosis (SARA) [5]. SARA disrupts microbial balance, reduces fermentation efficiency and microbial protein synthesis [6], and is associated with adverse outcomes such as decreased dry matter intake, diarrhea, milk fat depression, lameness, and, in severe cases, mortality [7]. Under intensive feeding systems, approximately 26% of dairy cattle are estimated to be at high risk of developing SARA [8].

Ruminal pH homeostasis is primarily regulated by VFA absorption and salivary buffering, which supplies bicarbonate and phosphate during rumination [9]. High-grain diets frequently exceed this buffering capacity, leading to acid accumulation and pronounced pH fluctuations [10]. Consequently, dietary buffer supplementation has been widely adopted to stabilize ruminal pH and reduce the severity of SARA [10,11]. Sodium bicarbonate, introduced in the 1960s, remains the most widely used ruminal buffer because of its effectiveness in neutralizing excess acidity, enhancing fiber digestion, stimulating microbial protein synthesis, and improving the utilization of fermentable organic matter [12]. Its supplementation also increases water intake, reduces ruminal osmotic pressure, and limits the proliferation of acid-tolerant bacteria such as lactobacilli [13]. Potassium-based buffers have more recently been proposed as alternative buffering agents with potential economic advantages [14]. In vitro studies demonstrate that buffer supplementation in high-concentrate TMRs increases ruminal pH and total VFA concentration, modifies VFA profiles, and reduces excessive butyrate accumulation under acidotic conditions [15,16]. These effects are particularly evident during rapid dietary transitions from high-forage to high-concentrate rations, when the risk of SARA is greatest [15,17].

Beyond its health consequences, SARA negatively affects nutrient utilization and feed efficiency. Controlled feeding studies report reduced dry matter intake, prolonged exposure to low ruminal pH, and decreased milk fat content in cows fed high-concentrate diets [8]. Improving feed digestibility is therefore a central objective in ration formulation and performance evaluation. Digestibility refers to the proportion of ingested feed that is retained and utilized by the animal [18,19]. Among available assessment techniques, the in vitro gas production method is widely used to characterize ruminal fermentation kinetics because it is cost-effective, reproducible, and conducted under controlled conditions [19,20]. This method is based on the principle that cumulative gas production, mainly carbon dioxide (CO_2_) and methane (CH_4_), is proportional to the extent of substrate fermentation under simulated ruminal conditions [20]. Diets with higher concentrate proportions typically produce greater gas volumes, reflecting increased microbial activity. A substantial proportion of CO_2_ originates from reactions between fermentation acids and bicarbonate in the incubation medium [21], and strong correlations have been reported between gas production and short-chain fatty acid concentrations [22].

Reliable interpretation of *in vitro* gas production data requires appropriate mathematical models to describe fermentation dynamics. Ruminal fermentation is a complex biological process with inherently nonlinear behavior, making nonlinear regression models particularly suitable for its analysis [23]. Buffer supplementation, including the addition of 1.0% sodium bicarbonate, has been shown to improve fiber digestibility under acidogenic conditions [24], with meta-analytical evidence indicating more consistent effects on forage digestibility than on total dry matter digestibility [25]. Because ruminal pH strongly influences fermentation kinetics and nutrient availability, modeling approaches must adequately capture the effects of dietary composition and buffering conditions. Enhancing feed digestibility, defined as the fraction of ingested feed retained and utilized by the animal, remains essential for improving feed efficiency and economic returns in dairy production systems [26].

The *in vitro* gas production technique is widely applied to evaluate feedstuffs, dietary additives [27], fermentation characteristics [28], and anti-nutritional factors [29]. Its reliability is supported by strong correlations with *in vivo* digestibility measurements [30]. When combined with nonlinear modeling, gas production data allow estimation of biologically relevant parameters such as potential gas production, degradation rate, and lag phase duration [23]. Previous studies have identified the time required to reach half of the maximum gas volume (t_1/2_) as a sensitive indicator of fermentation dynamics under varying forage levels [31,32]. Accurate modeling of cumulative gas production is therefore critical for evaluating the nutritional value of ruminant diets [33].

Numerous nonlinear models have been proposed to describe gas production kinetics; however, these models differ in their mathematical structure, underlying assumptions, and sensitivity to experimental conditions [34]. Exponential (EXP) and sigmoidal models are among the most commonly applied approaches [31,35,36]. Models such as the Fractional Rate Constant (FRC) and EXP assume proportionality between gas production rate and degradable substrate, whereas sigmoidal models, including Logistic (LOG) and Gompertz functions, incorporate microbial growth dynamics and exhibit fixed inflection points [37]. In contrast, modified Mitscherlich and Michaelis–Menten (MM) models lack fixed inflection points and may provide greater flexibility under certain fermentation scenarios [36]. Because dietary buffers and forage-to-concentrate ratios alter fermentation patterns, model performance is expected to vary across diets and incubation conditions. Accordingly, no single model can be assumed a priori to be universally superior or robust [34]. Comparative evaluation is therefore required to identify models that yield stable parameter estimates, low residual error, and biologically interpretable results across diverse nutritional contexts.

Despite limitations related to fitting assumptions and restricted capacity to incorporate non-temporal variables [38,39], nonlinear regression remains a fundamental tool for characterizing ruminal fermentation processes [39,40]. Recent studies emphasize the importance of integrating nutritional, biochemical, and mineral factors when modeling fermentation kinetics. Nonlinear models such as Michaelis–Menten and Mitscherlich have demonstrated strong predictive performance and revealed close associations between fermentation parameters, feed composition, microbial populations, and mineral content [41]. Comparative analyses across diverse feedstuffs indicate that generalized forms of Mitscherlich and Michaelis–Menten, as well as Gompertz and logistic models, offer greater flexibility for describing both sigmoidal and non-sigmoidal gas production profiles [42]. Moreover, in vitro gas production data can be used to derive effective first-order rate constants for ruminal carbohydrate digestion by integrating substrate degradation with passage kinetics, thereby providing a mechanistic framework for predicting fermentation dynamics under different dietary conditions [43].

The objective of this study was to identify suitable nonlinear regression models for describing ruminal fermentation kinetics using *in vitro* gas production data from diets differing in forage-to-concentrate ratio and dietary buffer supplementation. It was hypothesized that buffer inclusion would stabilize the ruminal environment and modify gas production patterns. Given the complexity of fermentation processes and dietary variability, no single model was expected to perform optimally under all conditions. Therefore, 32 nonlinear models reported in the literature were systematically compiled and evaluated for each diet–buffer combination. In addition to cumulative gas production, instantaneous digestion rates were derived from first-order derivatives of the fitted models to provide mechanistic insight into substrate-specific degradation kinetics. This integrated framework enables robust comparison of model performance, clarifies model limitations under buffered conditions, and offers practical guidance for model selection in applied ruminant nutrition research.

## Materials and methods

### Feed samples, buffers, and experimental design

Seven total mixed rations (TMRs) were formulated to represent different forage-to-concentrate ratios, primarily varying in neutral detergent fiber (NDF) content. The diets were designed to meet the nutritional requirements of mid-lactation Holstein cows producing 30 kg of milk per day, with an average body weight of 680 kg, according to NRC (2005) guidelines. The experimental treatments included a control diet (C) and high (HF), medium (MF), and low forage (LF) diets, each with or without an acidosis challenge (AC). The full ingredient composition and chemical characteristics of the diets are presented in Table 1.

**Table 1:** Ingredient composition and chemical characteristics (as % of dry matter) of experimental total mixed rations (TMRs) formulated with varying forage-to-concentrate ratios and the presence or absence of an acidosis challenge.

Ingredient/ Composition (%)	C-NoBuff	HF-NoBuff	MF-NoBuff	LF-NoBuff	HF-AC-NoBuff	MF-AC-NoBuff	LF-AC-NoBuff
Corn silage	18.93	17.29	15.91	14.74	13.83	12.73	11.79
Alfalfa hay	21.48	19.62	18.06	16.73	15.7	14.45	13.38
Corn grain	19.55	22.11	24.26	26.1	17.69	19.41	20.88
Barley grain	20.15	22.79	25.01	26.9	18.23	20.01	21.52
Soybean meal	9.79	8.94	8.23	7.62	7.15	6.58	6.1
Wheat bran	4.56	2.92	1.54	0.36	2.34	1.23	0.29
Rapeseed meal	5.54	6.33	6.99	7.55	5.06	5.59	6.04
Wheat starch	0	0	0	0	20	20	20
Crude protein	16.64	16.43	16.26	16.1	13.14	13	12.88
Neutral detergent fiber	40.44	38.71	37.26	36.03	30.97	29.81	28.82

All feed ingredients were oven-dried, ground using a Retsch Muhle mill (Model EPP 15X20, Retsch GmbH, Germany), and passed through a 1-mm screen before chemical analyses and *in vitro* gas production assays. Dry matter (DM) content was determined by drying samples in a forced-air oven at 95 °C for 24 hours. Nitrogen content was measured using the Kjeldahl method (Kjeltec 2300 Autoanalyzer, Foss Tecator AB, Höganäs, Sweden), and crude protein (CP) was calculated as N × 6.25.

To evaluate the effect of subacute ruminal acidosis, three buffering agents — sodium bicarbonate (NaHCO_3_), sodium carbonate (Na_2_CO_3_), and potassium carbonate (K_2_CO_3_) — were added to selected treatments. Each buffer was included at 1% of diet dry matter, following previous studies on ruminal pH stabilization. Abbreviations and codes for all combinations of forage level, acidosis challenge, and buffer supplementation are provided in Table 2.

**Table 2:** Abbreviations and treatment codes for experimental diets used in *in vitro* gas production trials, categorized by forage level, acidosis challenge status, and type of buffer supplementation.

Forage Level and Challenge Status	No Buffer	Sodium Bicarbonate	Sodium Carbonate	Potassium Carbonate
Control diet	C-NoBuff	C-SB	C-SC	C-PC
High forage (no challenge)	HF-NoBuff	HF-SB	HF-SC	HF-PC
Medium forage (no challenge)	MF-NoBuff	MF-SB	MF-SC	MF-PC
Low forage (no challenge)	LF-NoBuff	LF-SB	LF-SC	LF-PC
High forage (acidosis challenge)	HF-AC-NoBuff	HF-AC-SB	HF-AC-SC	HF-AC-PC
Medium forage (acidosis challenge)	MF-AC-NoBuff	MF-AC-SB	MF-AC-SC	MF-AC-PC
Low forage (acidosis challenge)	LF-AC-NoBuff	LF-AC-SB	LF-AC-SC	LF-AC-PC

### *In vitro* fermentation of substrates

*In vitro* fermentation was performed using seven diets, either without supplementation or with one of three dietary buffers: sodium bicarbonate, sodium carbonate, or potassium carbonate, each added at 1% of diet dry matter. For each treatment, 500 mg of air-dried and ground feed was placed into individual 120 mL gas-tight culture bottles. A total of 75 bottles were prepared, including three replicates per treatment and three blanks per treatment group to correct for gas produced by the inoculum alone.

The experimental procedure followed the *in vitro* gas production method described by Menke et al [30], using strict anaerobic techniques throughout rumen fluid collection, transfer, and incubation [22,44].

Before inoculation, each bottle was pre-warmed in a water bath at 39 °C for 1 hours. Then, 40 mL of buffered rumen inoculum was added under CO_2_ flushing. The inoculum was freshly prepared for each run by mixing filtered rumen fluid with a reduced mineral buffer medium in a 1:2 ratio (1 part rumen fluid to 2 parts medium), as described by [22].

The reduced medium (prepared in 3000 mL batches) contained:

- 375 mL distilled water
- 750 mL rumen buffer solution (35 g NaHCO_3_ and 4 g NH_4_HCO_3_ per liter)
- 750 mL rumen fluid from dairy steers
- 1125 mL macro-mineral solution (6.2 g KH_2_PO_4_, 5.7 g Na_2_HPO_4_, 2.22 g NaCl, and 0.6 g MgSO_4_·7H_2_O per liter)
- 0.30 mL micro-mineral solution (10 g MnCl_2_·4H_2_O, 13.2 g CaCl_2_·2H_2_O, 1 g CoCl_2_·6H_2_O, 8 g FeCl_3_·6H_2_O in 100 mL distilled water)
- 3.66 mL resazurin indicator (0.1 g in 100 mL distilled water)
- 125.1 mL freshly prepared reduction solution (120 mL distilled water, 3 g Na_2_S·9H_2_O, and 3 mL of 1 M NaOH)

The rumen fluid–medium mixture was continuously stirred under a CO_2_ stream and maintained at 39 °C using a magnetic stirrer with a heated base. Aliquots of 40 mL were dispensed into the pre-warmed bottles, which were then sealed with butyl rubber stoppers and aluminium crimp caps.

Bottles were incubated in a water bath at 39 °C, and cumulative gas production was monitored over a 96-hours incubation period to obtain a complete time-series dataset for each dietary treatment. Gas volumes were recorded at 1.5, 3, 6, 9, 24, 30, 48, 72, and 96 hour post-inoculation, with time zero defined as the baseline, using a pressure transducer coupled with a gas-tight syringe. Each diet × buffer combination was incubated in triplicate, producing three independent gas production curves per treatment. In each run, three blank bottles containing inoculum without substrate were included to correct gas production values for inoculum-derived gas.

### Nonlinear regression modeling of gas production kinetics

Nonlinear regression was used to describe the fermentation kinetics of the different total mixed rations (TMRs) under various buffering treatments. A total of 32 models (Table 3) were selected to cover a wide range of theoretical and empirical approaches commonly applied in ruminal fermentation studies. These models capture both sigmoidal and asymptotic patterns, account for lag phases or inflection points, and allow for one- or two-pool substrate degradation kinetics.

**Table 3:** Nonlinear mathematical models used to describe the cumulative gas production (y, mL) as a function of incubation time (x, h) across different TMRs and buffer treatments.

Model	Name	Category	Form
*M1*	Logistic-Exponential without LAG *(LE0)*	SIG	$y=(\frac{b\text{1}\times (\text{1}-\text{exp}(-b\text{2}\times \text{x}))}{\left(\text{1}+\text{exp}\left(\text{log}\frac{\text{1}}{b\text{3}}\right)-b\text{2}\times \text{x}))\right)}$
*M2*	Logistic-Exponential with LAG *(LE LAG)*	SIG	$y=(\frac{b\text{1}\times (\text{1}-\text{exp}(-b\text{2}\;\times (\text{x1}-\text{b3})))}{\left(\text{1}+\text{exp}\left(\;\text{log }(\frac{\text{1}}{b\text{4}}\right)-\;b\text{2}\;\times (\text{x1}-\text{b3}))\right)}$
*M3*	Exponential model without LAG *(EXP0)*	SEM	y=(b1 ×(1−exp(b2)×x))
*M4*	Exponential model with LAG *(EXP Lag)*	SEM	y=(b1 ×(1−exp(b2)×(x−b3)))
*M5*	Gompertz model	SIG	y=(b1 ×exp(−1 ×exp(1−b2)×(x−b3)))
*M6*	Logistic model *(LOG)*	SIG	$y=(\frac{b\text{1}}{(\text{1}+\text{exp}\left(\text{2}+b\text{2}\times (\text{b3}-\text{x}))\right)})$
*M7*	Generalization of the Mitscherlich	EMP	$y=(\text{b1}\times \left(\text{1}-\text{exp}\left(-\text{b2 }\times (\text{x}-\text{b3})-\text{b4}\times \left(x^{\text{0}.\text{5}}\;-\;{b\text{3}}^{\text{0}.\text{5}}\;\right)\right)\right))$
*M8*	Michaelis-Menten *(MM)*	EKM	$y=(\frac{b\text{1}\;\times \;x^{b\text{2}}}{\left(x^{b\text{2}}+{b\text{3}}^{b\text{2}}\right)})$
*M9*	Modified MM *(MMM)*	EKM	$y=(\frac{b\text{1}\;\times \;x^{b\text{2}}}{\left(x^{b\text{2}}+b\text{3}\right)})$
*M10*	Two-pool exponential *(TPEXP)*	DPM	y=(b1×(1−exp(−b2×(x−b3)))+b4×(1−exp(b5×(x−b3))))
*M11*	Two-pool logistic *(TPLOG)*	DPM	$y=\left(\frac{b\text{1}}{\left(\text{1}+\text{exp}\left(\text{2}-\text{4}\times \text{b2}\times (\text{x}-\text{b3})\right)\right)}\right)+\left(\frac{b\text{4}}{\left(\text{1}+\text{exp}\left(\text{2}-\text{4}\times \text{b5}\times (\text{x}-\text{b3})\right)\right)}\right)$
*M12*	Modified Gompertz model *(MGOM)*	SIG	$y=(\text{b1}\times \text{exp}\left(-\text{1}\times \text{exp}\left(\left(\frac{b\text{2}\times \text{2}.\text{7183}}{b\text{1}}\right)\times (\text{b3}-\text{x})+\text{1}\right)\right))$
*M13*	Logistic model *(LOG2)*	SIG	$y=(\frac{b\text{1}\;}{\left(\text{1}+b\text{2}\times \text{exp}(\text{b3}-\text{x})\right)})$
*M14*	Gompertz model *(GOM2)*	SIG	y=(b1×exp(−b2×exp(−b3×x)))
*M15*	Richard model *(RCD)*	EMP	$y=(\frac{b\text{1}\;}{(\text{1}+b\text{2}\times {\text{exp}(-b\text{3}\times \text{x}))}^{\left(\frac{\text{1}}{b\text{4}}\right)}})$
*M16*	Double-Sigmoid Model *(DSM)*	EMP	$y=(\frac{b\text{1}}{\left(\text{1}+\text{exp}\left(-(b\text{2}+b\text{3}\times \text{x}+\text{b4}\times x^{\text{2}}+b\text{5}\times x^{\text{3}}\right))\right)})$
*M17*	Monomolecular- logisticmodel *(MLM)*	EMP	$y=(\frac{b\text{1}\times \left(\text{1}-\text{exp}(-\text{b2}\times \text{x})\right)+\text{b3}}{\left(\text{1}+\text{exp}(-b\text{4}\times (\text{x}-\text{b5}))\right)})$
*M18*	Chapman-Richard model *(CRM)*	EMP	$y=(b\text{1}\times {\left(\text{1}-b\text{2}\times \text{exp}(-\text{b3}\times \text{x})\right)}^{\left(\frac{\text{1}}{\left(\text{1}-b\text{4}\right)}\right)})$
*M19*	Exponential-linear model *(ELM)*	EMP	$y=(\frac{b\text{1}\;}{\left(b\text{2}\times \text{log}\left(\text{1}+\text{exp}\left(\text{b2}\times (\text{x}-\text{x}\times \text{b3})\right)\right)\right)})$
*M20*	Exponential-linear model *(ELM)*	EMP	$y=(\text{b1}\times \text{log}(\text{exp}\left(\frac{b\text{2}\times (\text{x}-\text{b3})}{b\text{4}}\right)+\text{exp}\left(\frac{b\text{5}\times (\text{x}-\text{b6})}{b\text{7}}\right))+b\text{6}))$
*M21*	Contois model	EMP	$y=(\text{b1}\times \left(\frac{\text{1}-b\text{2}}{\left(b\text{3}\times \text{x}+\text{b2}-\text{1}\right)}\right))$
*M22*	France model 1	EMP	y=a ×(1−exp(−b ×( x−c)−d ×(sqrt (x)−sqrt (c))))
*M23*	France model *2*	EMP	$y=\frac{(a\;\times \left(\;\text{1}-\text{exp}(-\text{b }\times \text{x})\right))\;}{\left(\text{1}-c\;\times \text{exp}(-\text{b}\times \text{x})\right)}$
*M24*	Schnute model *1*	EMP	$y=({a\text{1}}^{b}+({a\text{2}}^{b}-{a\text{1}}^{b}\times {\left(\;\frac{\left(\text{1}-\text{exp}\left(-\text{a3 }\times (\text{x}-\text{c1})\right)\right)}{\left(\text{1}-\text{exp}\left(-a\text{3}\times (\text{c2}-\text{c1})\right)\right)}\right)}^{\left(\frac{\text{1}}{b}\right)}$
*M25*	Schnute model *2*	EMP	$y=\left({\left(\text{b1})+\text{b2 }\times \text{exp}(\text{b3}\times \text{x})\right)}^{b\text{4}}\right)\;$
*M26*	Monod model without LAG	EKM	$y=\left(\frac{b\text{1}\times \text{b2}\times \text{x}}{\left(b\text{2}\times \text{x}+\text{1}\right)}\right)$
*M27*	Monod model with LAG	EKM	$y=(\frac{b\text{1}\times \text{b2}\times (\text{x}-\text{b3})}{\left(b\text{3}\times (\text{x}-\text{b3})+\text{1}\right)})$
*M28*	one pool Gompertz function	SIG	y=b1×exp(−1×exp(1+b2×exp1×(b3−x)))
*M29*	Two-pool Gompertz function	DPM	y=b1×exp(−1×exp(1+b2×(b3−x))+b4×exp(1+b5×(b6−x)))
*M30*	first-order kinetic model of Ørskov and McDonald	SEM	y=(b1+b2×(1−exp(−b3×x)))
*M31*	Maxwell 1	SEM	$y=(b\text{1}+b\text{2}\times \text{exp}\left(\frac{-x}{b\text{3}}\right))$
*M32*	Maxwell 2	SEM	$y=b\text{1}+b\text{2}\times \text{exp}\left(\frac{-x}{b\text{3}}\right)+\text{exp}\left(\frac{-x}{b\text{5}}\right)$

**Note:** SEM = Simple Exponential Models; SIG = Sigmoidal Models; DPM = Dual-Pool Models; EKM = Enzyme-Kinetic Models; EMP = Empirical/Flexible Models.

Nonlinear regression was used to describe the relationship between fermentation time (x, h) and cumulative gas volume (y, mL) by fitting models of the form:


$$\begin{array}{c} y_{i}=f\left(x_{i};\theta \right)+\varepsilon_{i},\;i=\text{1},\text{2},\ldots ,n \end{array}$$ (1)

where $y_{i}$ is the observed gas production at time $x_{i}$, $f(x_{i};\theta )$ is the predicted value based on the parameter vector θ, and $\varepsilon_{i}$ represents the residual error. Each parameter in θ has a biological interpretation, such as asymptotic gas volume (A), fractional fermentation rate constant (k), or lag time (λ), depending on the chosen model. Residuals were assumed to be independent and identically distributed with a mean of zero and either constant or structured variance.

Model parameters were estimated by minimizing the residual sum of squares (RSS):


$$\begin{array}{c} RSS(\theta )=\sum {\left(f\left(x_{i};\theta \right)-y_{i}\right)}^{\text{2}} \end{array}$$ (2)

This minimization was performed using iterative optimization algorithms, primarily Levenberg–Marquardt or trust-region methods. Appropriate initial parameter estimates were critical to avoid convergence to local minima, especially for complex models.

The 32 candidate models (Table 3) were classified into five categories:

- Simple Exponential Models (SEM): One-pool, first-order kinetics assuming immediate fermentation onset and single-fraction substrate degradation. Models: M3 (EXP0), M4 (EXP Lag), M30 (Ørskov & McDonald), M31 (Maxwell 1), M32 (Maxwell 2).
- Sigmoidal Models (SIG): Capturing an initial lag phase followed by accelerated gas production and a final asymptote. Models: M5 (Gompertz), M6 (Logistic), M12 (Modified Gompertz), M13 (Logistic 2), M14 (Gompertz 2), M15 (Richards).
- Dual-Pool Models (DPM): Partitioning substrate into fast- and slow-degrading fractions, each with its own rate constant. Models: M10 (Two-pool exponential), M11 (Two-pool logistic), M29 (Two-pool Gompertz).
- Enzyme-Kinetic Models (EKM): Describing gas production as a substrate-limited saturation process. Models: M8 (Michaelis–Menten), M9 (Modified MM), M26 (Monod without LAG), M27 (Monod with LAG).
- Empirical/Flexible Models (EMP): Providing adjustable inflection points and flexible curvature control. Models: M1 (LE0), M2 (LE LAG), M7 (Generalized Mitscherlich), M16 (Double-Sigmoid), M17 (Monomolecular-logistic), M18 (Chapman-Richard), M19–M20 (Exponential-linear), M21 (Contois), M22–M23 (France models), M24–M25 (Schnute), M28 (One-pool Gompertz).

Each model was independently fitted to the time-series gas production data for all diet-buffer combinations. To improve robustness against outliers and heteroscedasticity, robust nonlinear least-squares fitting was applied using MATLAB (*The MathWorks Inc., Natick, Massachusetts, USA; version R2025a*). M-estimator weighting functions (e.g., Andrews, Huber, Cauchy) were tested, and different error structures—constant, proportional, and combined—were evaluated to improve residual homogeneity and model accuracy. Parallel computation and optimized convergence settings ensured computational efficiency and reliable parameter estimation across all treatments.

Model performance was evaluated using four statistical metrics: the coefficient of determination (R²), adjusted R², sum of squared errors (SSE), and root mean squared error (RMSE). To enable direct comparison, each metric was normalized to a [0,1] scale using min–max normalization:


$$\begin{array}{c} {NM}_{i}=\frac{M_{i}-\text{min}(M)}{\text{max}(M)-\text{min}(M)} \end{array}$$ (3)

where $M_{i}$ is the value of the metric for the i-th model, and min (M)  and max (M) are the minimum and maximum values of that metric across all models. For error metrics (SSE and RMSE), which decrease with better model performance, the normalized scores were inverted:


$$\begin{array}{c} {NE}_{i}=\text{1}-{NM}_{i}\; \end{array}$$ (4)

A composite performance score ($S_{i}$) was then calculated for each model as a weighted sum of the normalized metrics:


$$\begin{array}{c} \;S_{i}=w_{\text{1}}\overset{\text{2}}{\underset{Norm}{R}}+w_{\text{2}}\overset{\text{2}}{\underset{Adj.Norm}{R}}+w_{\text{3}}{SSE}_{Norm}+w_{\text{4}}{RMSE}_{Norm}\; \end{array}$$ (5)

where $w_{\text{1}},w_{\text{2}},w_{\text{3}},$ and $w_{\text{4}}\;$ are the weights assigned to each metric. Equal weights of 0.25 were applied in this study to ensure balanced consideration. The resulting composite scores allowed objective ranking and selection of the best-performing model for each dietary treatment

## Results and discussion

The results of the nonlinear regression analyses are presented below. First, model fit statistics for all 32 candidate functions are reported for each dietary treatment. Key performance metrics are compared to identify the models that best describe gas production under different forage-to-concentrate ratios and buffer supplementation regimes. Next, the effects of buffer inclusion on fermentation kinetics are evaluated, focusing on fermentation efficiency, lag-phase duration, and potential impacts on volatile fatty acid (VFA) production. The findings are interpreted in the context of strategies to mitigate subacute ruminal acidosis.

### Ranking of nonlinear models across dietary treatments

The performance of nonlinear models was systematically evaluated across dietary treatments and buffering conditions using the composite performance score defined in the Materials and Methods. Given the large number of candidate models (n = 32) and experimental combinations (n = 28 diet–buffer treatments), model assessment was performed in two sequential steps: an initial quantitative screening based on statistical performance metrics, followed by a biological and statistical appraisal of the highest-ranked models.

For clarity and conciseness, only the top 10 models for each dietary treatment are presented in Table 4. Because these models represent a preselected subset of the best-performing candidates, their composite performance scores were expectedly close. The narrow range of values observed (generally 0.97–1.00) therefore reflects the high explanatory power of the selected models rather than insufficient discrimination by the evaluation metric. Under the control diet (C), the Michaelis–Menten model (Model 8) consistently ranked first across all buffering treatments (NoBuff, SB, SC, and PC). This result indicates that a saturation-type kinetic function adequately describes gas production dynamics in the absence of dietary or acidotic stress, where substrate availability is the dominant limiting factor. In high-forage diets without acidosis (HF), the exponential model (Model 1) and the Michaelis–Menten model alternated as the top-ranked functions. This pattern suggests that both first-order degradation and saturable kinetics can satisfactorily represent fermentation processes in fiber-rich rations. When acidosis was induced (HF-AC), the Michaelis–Menten model again achieved the highest rank, demonstrating its robustness under reduced ruminal pH and altered fermentation conditions. For medium-forage diets without challenge (MF), the Michaelis–Menten model consistently ranked first across all buffering treatments, confirming its suitability for describing fermentation kinetics in moderately fibrous diets. Under acidosis challenge (MF-AC), top rankings alternated primarily between the Michaelis–Menten and exponential models, indicating that both saturation effects and first-order kinetics contribute to gas production when fiber level and acid stress interact. In low-forage, high-concentrate diets without challenge (LF), the exponential and Michaelis–Menten models provided the best fits, consistent with rapid fermentation of readily fermentable carbohydrates. Under acidotic conditions (LF-AC), the Michaelis–Menten and logistic models (Model 3) frequently shared the highest ranks. This outcome suggests that asymmetric sigmoidal behavior becomes increasingly relevant when rapid acid accumulation modifies the lag phase and fermentation rate. Across all dietary treatments, buffer type (SB, SC, or PC) did not alter the identity of the top-ranked model. However, minor shifts in secondary rankings (e.g., Models 2, 4, 28, and 29) reflected subtle buffer-related effects on fermentation dynamics.

**Table 4:** Ranking of the top-selected models among 30 candidates for different dietary treatments, based on performance scores.

Diet	No buff		SB		SC		PC	
	Model	Performance	Model	Performance	Model	Performance	Model	Performance
Control diet	M8	1.00	M8	1.00	M8	1.00	M8	1.00
	M1	1.00	M1	1.00	M7	1.00	M29	1.00
	M3	0.99	M2	1.00	M1	1.00	M28	1.00
	M28	0.99	M12	0.99	M4	1.00	M4	1.00
	M4	0.99	M26	0.99	M28	1.00	M3	1.00
	M29	0.99	M3	0.99	M17	1.00	M1	1.00
	M2	0.99	M22	0.99	M3	1.00	M7	1.00
	M22	0.99	M4	0.99	M10	1.00	M2	1.00
	M10	0.99	M28	0.99	M21	0.99	M21	1.00
	M17	0.99	M29	0.99	M24	0.99	M10	0.99
High forage diet (no acidosis challenge)	M1	1.00	M1	1.00	M8	1.00	M8	1.00
	M8	1.00	M8	1.00	M7	1.00	M24	1.00
	M2	1.00	M28	0.99	M28	1.00	M3	0.99
	M15	1.00	M29	0.99	M29	1.00	M29	0.99
	M3	1.00	M4	0.99	M4	1.00	M4	0.99
	M22	1.00	M2	0.99	M1	1.00	M28	0.99
	M29	1.00	M3	0.99	M2	1.00	M1	0.99
	M4	1.00	M7	0.99	M3	1.00	M22	0.99
	M28	1.00	M22	0.99	M22	1.00	M21	0.99
	M24	0.99	M24	0.99	M17	0.99	M25	0.99
Medium forage diet (no acidosis challenge)	M8	1.00	M8	1.00	M8	1.00	M24	1.00
	M1	1.00	M3	1.00	M29	1.00	M8	1.00
	M2	0.99	M1	1.00	M28	1.00	M22	1.00
	M28	0.99	M22	1.00	M4	1.00	M29	1.00
	M29	0.99	M28	1.00	M1	1.00	M28	1.00
	M4	0.99	M4	1.00	M2	0.99	M4	1.00
	M3	0.99	M29	1.00	M3	0.99	M1	0.99
	M10	0.99	M2	1.00	M22	0.99	M3	0.99
	M22	0.99	M10	1.00	M10	0.99	M21	0.99
	M21	0.99	M24	0.99	M12	0.98	M25	0.99
Low forage diet (no acidosis challenge)	M1	1.00	M3	1.00	M8	1.00	M8	1.00
	M2	1.00	M8	1.00	M1	1.00	M24	1.00
	M8	1.00	M22	1.00	M2	1.00	M21	1.00
	M7	0.99	M29	1.00	M7	1.00	M3	1.00
	M28	0.99	M4	1.00	M4	1.00	M25	0.99
	M29	0.99	M1	1.00	M29	1.00	M1	0.99
	M4	0.99	M7	1.00	M28	1.00	M4	0.99
	M3	0.99	M2	1.00	M3	0.99	M28	0.99
	M22	0.99	M24	1.00	M22	0.99	M29	0.99
	M10	0.99	M10	1.00	M10	0.99	M22	0.99
High forage diet (acidosis challenge)	M8	1.00	M8	1.00	M8	1.00	M8	1.00
	M7	1.00	M1	1.00	M2	0.99	M29	1.00
	M1	1.00	M7	0.99	M1	0.99	M1	1.00
	M2	1.00	M2	0.99	M28	0.99	M4	1.00
	M4	0.99	M3	0.99	M4	0.99	M21	0.99
	M28	0.99	M28	0.99	M17	0.99	M24	0.99
	M29	0.99	M29	0.99	M10	0.99	M3	0.99
	M10	0.99	M4	0.99	M3	0.99	M25	0.99
	M3	0.99	M22	0.99	M22	0.99	M22	0.99
	M22	0.99	M10	0.99	M21	0.99	M2	0.99
Medium forage diet (acidosis challenge)	M8	1.00	M8	1.00	M1	1.00	M3	1.00
	M1	0.97	M1	1.00	M8	1.00	M8	1.00
	M2	0.97	M3	1.00	M2	1.00	M22	1.00
	M3	0.97	M29	1.00	M7	0.99	M28	1.00
	M22	0.97	M4	1.00	M3	0.99	M29	1.00
	M4	0.97	M28	1.00	M29	0.99	M4	1.00
	M29	0.97	M2	1.00	M4	0.99	M2	1.00
	M28	0.97	M22	1.00	M28	0.99	M1	1.00
	M21	0.97	M10	1.00	M22	0.99	M15	1.00
	M24	0.97	M17	1.00	M10	0.99	M10	0.99
Low forage diet (acidosis challenge)	M3	1.00	M1	1.00	M8	1.00	M8	1.00
	M8	1.00	M8	1.00	M1	1.00	M28	1.00
	M22	1.00	M2	1.00	M7	1.00	M4	1.00
	M1	1.00	M3	1.00	M2	0.99	M1	1.00
	M28	1.00	M4	1.00	M29	0.99	M3	1.00
	M4	1.00	M28	1.00	M4	0.99	M7	1.00
	M24	1.00	M17	1.00	M28	0.99	M22	1.00
	M2	1.00	M22	1.00	M26	0.99	M10	1.00
	M25	1.00	M21	1.00	M12	0.99	M21	1.00
	M17	0.99	M24	0.99	M10	0.99	M24	1.00

Although the composite performance score provided an effective framework for organizing and screening a large volume of results, final model selection was not based solely on numerical ranking. Additional criteria were applied, including (i) statistical significance of all model parameters at the 1% or 5% level, (ii) consistency of performance across dietary treatments, and (iii) model parsimony. Considering these combined statistical and biological criteria, the Michaelis–Menten model (Model 8) was selected as the primary function for subsequent comparative analyses.

### Evaluation of the Michaelis–Menten model across dietary treatments

The performance of the selected nonlinear model (Model 8; Michaelis–Menten) across dietary compositions and buffering conditions is summarized in Table 5. Model evaluation was based on the R², $\overset{\text{2}}{\underset{Adj.}{R}}$, and RMSE (mL gas), which collectively quantify explained variance, goodness of fit, and absolute prediction error.

**Table 5:** Performance metrics of the selected nonlinear model (Michaelis–Menten; Model 8) in predicting gas production kinetics under varying dietary and buffer conditions.

Treatment code	R^2^	$\overset{2}{\underset{Adj.}{R}}$	RMSE (mL)	Mean (mL)	Treatment code	R^2^	$\overset{2}{\underset{Adj.}{R}}$	RMSE (mL)	Mean (mL)
C-NoBuff	1.00	1.00	7.16	77.10	HF-AC-NoBuff	1.00	1.00	5.34	86.32
C-SB	0.98	0.98	10.53	81.18	HF-AC-SB	1.00	1.00	7.57	86.82
C-SC	1.00	1.00	3.46	68.01	HF-AC-SC	1.00	1.00	3.83	83.81
C-PC	1.00	0.99	5.43	76.74	HF-AC-PC	0.99	0.99	9.41	93.34
HF-NoBuff	0.99	0.99	10.11	88.80	MF-AC-NoBuff	0.99	0.99	9.65	90.99
HF-SB	0.99	0.99	9.70	89.12	MF-AC-SB	1.00	1.00	4.67	85.28
HF-SC	1.00	1.00	4.69	79.73	MF-AC-SC	1.00	1.00	5.71	84.86
HF-PC	0.99	0.99	14.15	84.51	MF-AC-PC	0.98	0.98	9.97	86.76
MF-NoBuff	0.99	0.99	6.83	78.93	LF-AC-NoBuff	0.98	0.98	10.97	79.10
MF-SB	0.99	0.99	8.45	84.28	LF-AC-SB	1.00	1.00	5.95	82.35
MF-SC	0.99	0.99	9.62	84.55	LF-AC-SC	0.99	0.99	7.56	82.90
MF-PC	0.99	0.99	10.82	88.63	LF-AC-PC	1.00	1.00	5.28	80.33
LF-NoBuff	0.99	0.99	9.35	82.38					
LF-SB	0.97	0.97	15.18	97.80					
LF-SC	0.99	0.99	7.46	85.91					
LF-PC	0.99	0.99	8.15	85.62					

Note: Treatment codes combine forage level and challenge status with buffer type: C, HF, MF, and LF denote control, high-, medium-, and low-forage diets; AC indicates acidosis challenge; NoBuff, SB, SC, and PC represent no buffer, sodium bicarbonate, sodium carbonate, and potassium carbonate, respectively.

Across all diets and buffering treatments, Model 8 consistently produced very high R² and $\overset{\text{2}}{\underset{Adj.}{R}}$ values (0.97–1.00), indicating that the model accurately captured the overall kinetics and temporal patterns of cumulative gas production. Interpretation of RMSE values was performed with explicit consideration of the response scale. In this study, cumulative gas production ranged from approximately 165–248 mL, with mean values typically between 68 and 98 mL, depending on dietary composition and buffer type. Accordingly, RMSE values between 3 and 15 mL corresponded to relative deviations of approximately 4–15% of the mean gas production, which is consistent with the expected biological variability of in vitro fermentation systems. Under the control diet, Model 8 exhibited near-perfect explanatory performance, with R² and $\overset{\text{2}}{\underset{Adj.}{R}}$ values equal to 1.00 for most buffering treatments. RMSE values were low relative to the mean gas production (e.g., 3.46–5.43 mL vs. 68–81 mL), indicating minimal absolute deviation between observed and predicted values and accurate representation of baseline fermentation kinetics. In high-forage diets without acidosis challenge, strong predictive performance was maintained across all buffers (R² = 0.99–1.00). RMSE values ranged from 4.69 to 14.15 mL, corresponding to relative errors of approximately 5–17% of mean gas production (≈80–90 mL). The lowest RMSE was observed with sodium carbonate buffering, suggesting more stable fermentation dynamics under this condition. When acidosis was induced, RMSE values decreased for the no-buffer and sodium carbonate treatments (3.83 and 5.34 mL, respectively), despite comparable or higher mean gas production, indicating robust model performance under acid-stressed conditions. For medium-forage diets, Model 8 maintained high explanatory accuracy both without and with acidosis challenge (R² = 0.98–1.00). RMSE values ranged from 4.67 to 10.82 mL, corresponding to relative deviations of approximately 5–13% of the mean gas production. Sodium bicarbonate and sodium carbonate buffers under acidosis challenge produced the lowest RMSE values, reflecting improved predictive precision under moderate fermentation stress. In low-forage, high-concentrate diets, RMSE values were generally higher, particularly under sodium bicarbonate buffering without acidosis (RMSE = 15.18 mL). This increase coincided with higher mean gas production (≈98 mL) and increased biological variability rather than deterioration of model structure, as evidenced by consistently high R² values (≥0.97). Under acidosis challenge, RMSE values declined to 5.28–10.97 mL across buffers, confirming effective modeling of rapidly fermentable substrates under pronounced pH perturbations.

Overall, the Michaelis–Menten model demonstrated high generalizability across dietary compositions and fermentation conditions. The combination of very high R² values and moderate RMSE values indicates that the model accurately represents fermentation kinetics while operating within the expected absolute error range imposed by the scale and inherent variability of gas production data. These findings support the selection of Model 8 as the preferred framework for comparative kinetic analyses in the present study.

### Estimated parameters of the Michaelis–Menten model

The estimated parameters of the Michaelis–Menten (MM) model—b_1_ (maximum response), b_2_ (Michaelis constant), and b_3_ (baseline activity)—varied across dietary treatments and buffer types (Tables 6–8), providing mechanistic insight into fermentation kinetics under different nutritional and physiological conditions.

**Table 6:** Estimated b₁ coefficients of the Michaelis–Menten model across dietary treatments, including 95% confidence intervals, standard errors, and significance levels.

Diet	Buffer	Lower 95% CI	Estimate	Upper 95% CI	SE	p-value
Control diet	No buff	169.89	190.3	210.72	9.95	< 1.0 × 10^−3^
	SB	170.68	194	217.31	11.36	< 1.0 × 10^−3^
	SC	180.61	192.83	205.04	5.95	< 1.0 × 10^−3^
	PC	178.72	196.07	213.42	8.46	< 1.0 × 10^−3^
High forage diet (no acidosis challenge)	No buff	203.96	235.34	266.71	15.29	< 1.0 × 10^−3^
	SB	203.19	246.88	290.56	21.29	< 1.0 × 10^−3^
	SC	196.1	212.22	228.33	7.86	< 1.0 × 10^−3^
	PC	148.48	196.97	245.47	23.64	< 1.0 × 10^−3^
Medium forage diet (no acidosis challenge)	No buff	182.48	199.97	217.46	8.52	< 1.0 × 10^−3^
	SB	218.65	267.44	316.24	23.78	< 1.0 × 10^−3^
	SC	162.77	183.9	205.03	10.3	< 1.0 × 10^−3^
	PC	220.71	300.56	380.4	38.91	< 1.0 × 10^−3^
Low forage diet (no acidosis challenge)	No buff	186.08	213.37	240.65	13.3	< 1.0 × 10^−3^
	SB	193.38	235.61	240.65	20.58	< 1.0 × 10^−3^
	SC	209.26	235.09	260.92	12.59	< 1.0 × 10^−3^
	PC	208.28	244.93	281.58	17.86	< 1.0 × 10^−3^
High forage diet (acidosis challenge)	No buff	207.56	222.01	236.47	7.04	< 1.0 × 10^−3^
	SB	210.29	229.41	248.54	9.32	< 1.0 × 10^−3^
	SC	208.86	219.22	229.59	5.05	< 1.0 × 10^−3^
	PC	209.38	244.35	279.33	17.04	< 1.0 × 10^−3^
Medium forage diet (acidosis challenge)	No buff	188.63	217.97	247.31	14.3	< 1.0 × 10^−3^
	SB	242.89	265.87	288.84	11.2	< 1.0 × 10^−3^
	SC	229.43	253.43	277.42	11.69	< 1.0 × 10^−3^
	PC	196.58	234.38	272.18	18.42	< 1.0 × 10^−3^
Low forage diet (acidosis challenge)	No buff	188.05	242.82	297.59	26.69	< 1.0 × 10^−3^
	SB	260.81	307.03	353.26	22.53	< 1.0 × 10^−3^
	SC	179.17	195.04	210.9	7.73	< 1.0 × 10^−3^
	PC	242.9	278.5	314.11	17.35	< 1.0 × 10^−3^

Note: No Buff = No Buffer; SB = Sodium Bicarbonate; SC = Sodium Carbonate; PC = Potassium Carbonate; CI = Confidence Interval; SE = Standard Error.

**Table 7:** Estimated b_2_ coefficients of the Michaelis–Menten model across dietary treatments, including 95% confidence intervals, standard errors, and significance levels.

Diet	Buffer	Lower 95% CI	Estimate	Upper 95% CI	SE	p-value
Control diet	No buff	1.17	1.41	1.65	0.12	< 1.0 × 10^−3^
	SB	1.1	1.41	1.72	0.15	< 1.0 × 10^−3^
	SC	1.22	1.34	1.46	0.06	< 1.0 × 10^−3^
	PC	1.08	1.24	1.39	0.08	< 1.0 × 10^−3^
High forage diet (no acidosis challenge)	No buff	1.06	1.31	1.57	0.13	< 1.0 × 10^−3^
	SB	0.92	1.16	1.39	0.11	< 1.0 × 10^−3^
	SC	1.11	1.24	1.37	0.06	< 1.0 × 10^−3^
	PC	0.81	1.22	1.64	0.2	< 1.0 × 10^−3^
Medium forage diet (no acidosis challenge)	No buff	1.2	1.41	1.61	0.1	< 1.0 × 10^−3^
	SB	0.91	1.12	1.32	0.1	< 1.0 × 10^−3^
	SC	1.08	1.37	1.65	0.14	< 1.0 × 10^−3^
	PC	0.84	1.09	1.33	0.12	< 1.0 × 10^−3^
Low forage diet (no acidosis challenge)	No buff	1.08	1.34	1.6	0.13	< 1.0 × 10^−3^
	SB	0.88	1.22	1.55	0.16	< 1.0 × 10^−3^
	SC	1.08	1.27	1.46	0.09	< 1.0 × 10^−3^
	PC	0.98	1.18	1.39	0.1	< 1.0 × 10^−3^
High forage diet (acidosis challenge)	No buff	1.25	1.4	1.55	0.07	< 1.0 × 10^−3^
	SB	1.24	1.44	1.64	0.1	< 1.0 × 10^−3^
	SC	1.32	1.44	1.55	0.05	< 1.0 × 10^−3^
	PC	0.99	1.21	1.44	0.11	< 1.0 × 10^−3^
Medium forage diet (acidosis challenge)	No buff	1.05	1.31	1.57	0.13	< 1.0 × 10^−3^
	SB	1.09	1.2	1.32	0.06	< 1.0 × 10^−3^
	SC	1.15	1.3	1.45	0.07	< 1.0 × 10^−3^
	PC	0.94	1.18	1.42	0.12	< 1.0 × 10^−3^
Low forage diet (acidosis challenge)	No buff	0.91	1.21	1.51	0.15	< 1.0 × 10^−3^
	SB	1.08	1.24	1.41	0.08	< 1.0 × 10^−3^
	SC	1.29	1.53	1.76	0.12	< 1.0 × 10^−3^
	PC	1.02	1.15	1.29	0.07	< 1.0 × 10^−3^

Note: No Buff = No Buffer; SB = Sodium Bicarbonate; SC = Sodium Carbonate; PC = Potassium Carbonate; CI = Confidence Interval; SE = Standard Error.

**Table 8:** Estimated b_3_ coefficients of the Michaelis–Menten model across dietary treatments, including 95% confidence intervals, standard errors, and significance levels.

Diet	Buffer	Lower 95% CI	Estimate	Upper 95% CI	SE	p-value
Control diet	No buff	20.75	26.1	31.46	2.61	< 1.0 × 10^−3^
	SB	15.27	20.38	25.48	2.49	< 1.0 × 10^−3^
	SC	25.89	29.46	33.03	1.74	< 1.0 × 10^−3^
	PC	19.92	24.55	29.18	2.26	< 1.0 × 10^−3^
High forage diet (no acidosis challenge)	No buff	18.84	25.78	32.71	3.38	< 1.0 × 10^−3^
	SB	19.34	30.93	42.52	5.65	< 1.0 × 10^−3^
	SC	22.22	26.4	30.58	2.04	< 1.0 × 10^−3^
	PC	12.38	25.87	39.37	6.58	< 1.0 × 10^−3^
Medium forage diet (no acidosis challenge)	No buff	19.49	23.58	27.67	1.99	< 1.0 × 10^−3^
	SB	22.79	36.9	51.01	6.88	< 1.0 × 10^−3^
	SC	14.48	19.26	24.03	2.33	< 1.0 × 10^−3^
	PC	19.76	43.89	68.01	11.76	< 1.0 × 10^−3^
Low forage diet (no acidosis challenge)	No buff	18.57	24.99	31.41	3.13	< 1.0 × 10^−3^
	SB	12.27	20.62	28.97	4.07	< 1.0 × 10^−3^
	SC	21.19	27.29	33.4	2.98	< 1.0 × 10^−3^
	PC	21.87	31.67	41.48	4.78	< 1.0 × 10^−3^
High forage diet (acidosis challenge)	No buff	21.53	24.68	27.82	1.53	< 1.0 × 10^−3^
	SB	20.16	24.05	27.93	1.89	< 1.0 × 10^−3^
	SC	23.13	25.42	27.72	1.12	< 1.0 × 10^−3^
	PC	19.22	27.49	35.77	4.03	< 1.0 × 10^−3^
Medium forage diet (acidosis challenge)	No buff	18.37	25.28	32.2	3.37	< 1.0 × 10^−3^
	SB	28.66	34.66	40.65	2.92	< 1.0 × 10^−3^
	SC	26.84	32.76	38.69	2.89	< 1.0 × 10^−3^
	PC	17.65	27.05	36.45	4.58	< 1.0 × 10^−3^
Low forage diet (acidosis challenge)	No buff	19.44	35.21	50.98	7.69	< 1.0 × 10^−3^
	SB	35.54	48.82	62.1	6.47	< 1.0 × 10^−3^
	SC	18.35	21.79	25.24	1.68	< 1.0 × 10^−3^
	PC	20.75	26.1	31.46	2.61	< 1.0 × 10^−3^

Note: No Buff = No Buffer; SB = Sodium Bicarbonate; SC = Sodium Carbonate; PC = Potassium Carbonate; CI = Confidence Interval; SE = Standard Error.

The b₁ coefficient, representing the theoretical maximum fermentation response, differed with forage level, acidosis challenge, and buffer type. Under the control diet, b₁ ranged from 190.3 without a buffer to 196.07 with potassium carbonate (PC), all with narrow confidence intervals and statistically significant differences (p < 0.001). In high-forage diets without acidosis, b₁ increased, particularly with sodium bicarbonate (SB, 246.88) and PC (196.97), indicating higher fermentation potential. The medium-forage diet without acidosis showed the largest b₁ increase, with PC yielding 300.56, suggesting markedly enhanced maximum fermentation activity. Under acidosis, all buffer treatments increased b₁ compared to the no-buffer control, with the low-forage diet and SB buffer reaching the highest value (307.03), highlighting the capacity of SB and PC to mitigate fermentation depression under acidic conditions.

The b_2_ coefficient, reflecting the substrate concentration at which the fermentation rate reaches half of its maximum, also showed treatment-specific patterns (Table 7). In the control diet, b_2_ values were relatively stable across buffers (1.24–1.44), with SC and PC showing slightly lower values, indicating higher substrate affinity. In high- and medium-forage diets without acidosis, SB led to consistently lower b_2_ values (e.g., 1.12 in medium forage), suggesting improved fermentation efficiency at lower substrate levels. Under acidosis, SC produced higher b_2_ values (e.g., 1.53 in low forage), reflecting its ability to maintain fermentation under acidic stress. All differences were statistically significant (p < 0.001).

The b_3_ parameter, representing basal fermentation activity (Table 8), showed more variability, particularly under acidosis. Without acidosis, SB increased b_3_ in medium-forage diets (36.90), whereas PC reached the highest value (43.89). Under acidosis, b_3_ was markedly elevated in low-forage diets with SB (48.82) and PC (31.46), indicating that these buffers help preserve or enhance basal fermentation. SC consistently produced moderate-to-high b_3_ values, particularly in acidosis-challenged medium-forage diets (32.76), demonstrating its stabilizing effect.

Overall, forage level and buffer supplementation significantly influenced MM parameters. SB and PC consistently enhanced maximum fermentation capacity (b_1_), improved substrate affinity (lower b_2_), and maintained basal activity (b_3_), especially under acidosis-prone conditions. These results support the strategic use of buffering agents to optimize rumen fermentation efficiency and reduce the negative effects of subacute ruminal acidosis.

### Fermentation kinetics across buffers and forage levels

**Fig 1:**
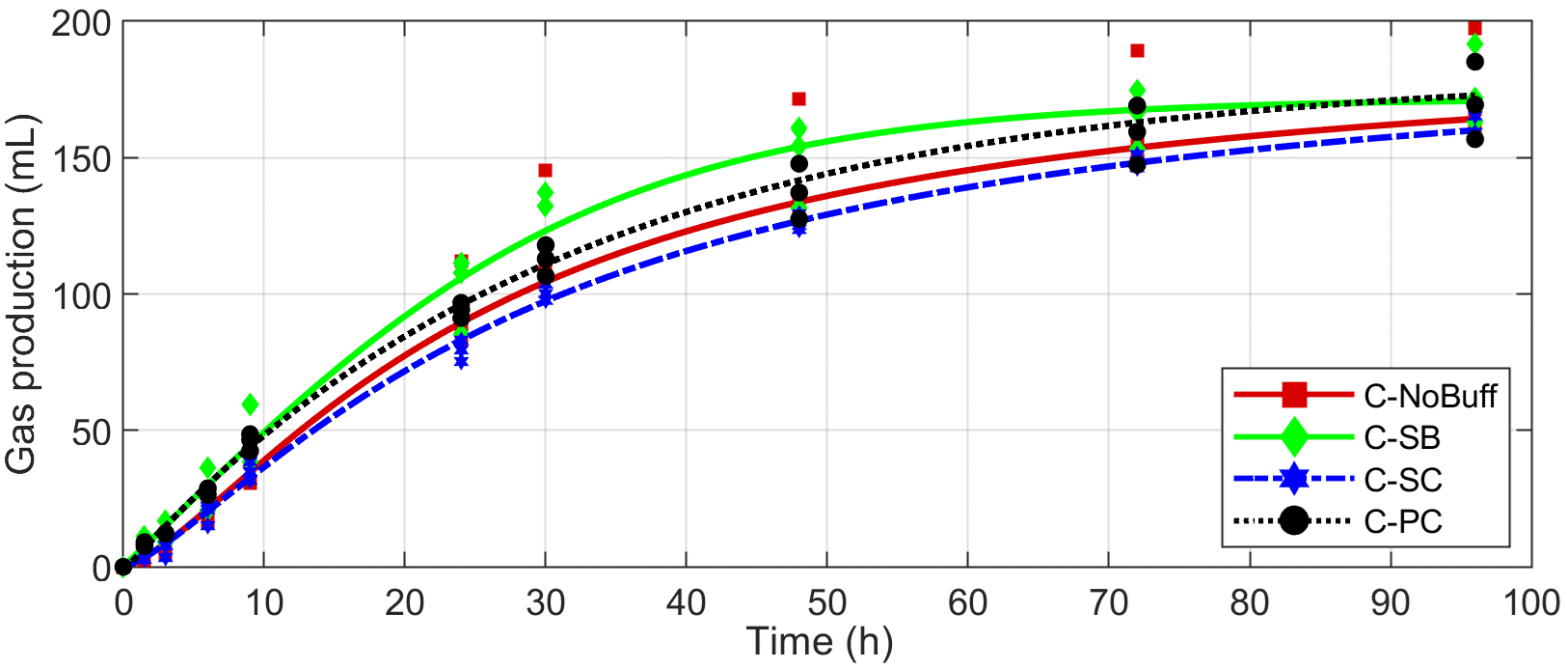
Predicted cumulative response of the Michaelis–Menten model for the control diet under four buffering treatments during a 96-hour acidosis challenge. (No buffer: C-NoBuff; Sodium bicarbonate: C-SB; Sodium carbonate: C-SC; Potassium carbonate: C-PC).

Fig 1 shows the temporal changes in the cumulative response predicted by the Michaelis–Menten model (Model 8) for the control diet under four buffering treatments during a 96-hour acidosis challenge. The unbuffered control (C-NoBuff) displayed the slowest increase, reaching about 164 units by 96 hours. In contrast, all buffer-supplemented treatments showed higher cumulative responses, indicating faster restoration of fermentation under acidotic conditions.

Among the buffers, sodium bicarbonate (C-SB) had the strongest early effect, with a steep rise during the first 6–8 hours and reaching around 171 units at the end of the challenge. Potassium carbonate (C-PC) followed a similar pattern, with a slight delay, attaining nearly 173 units by hour 96. Sodium carbonate (C-SC) provided an intermediate response, reaching approximately 160 units, indicating a moderate buffering effect. Differences in the response curves became noticeable after the initial 6–8 hours and persisted throughout the 96-hour period, highlighting the sustained ability of buffers to mitigate ruminal acidification.

Overall, buffer supplementation substantially accelerated the recovery of fermentation activity under acidosis, with sodium bicarbonate and potassium carbonate showing superior performance compared to sodium carbonate.

**Fig 2:**
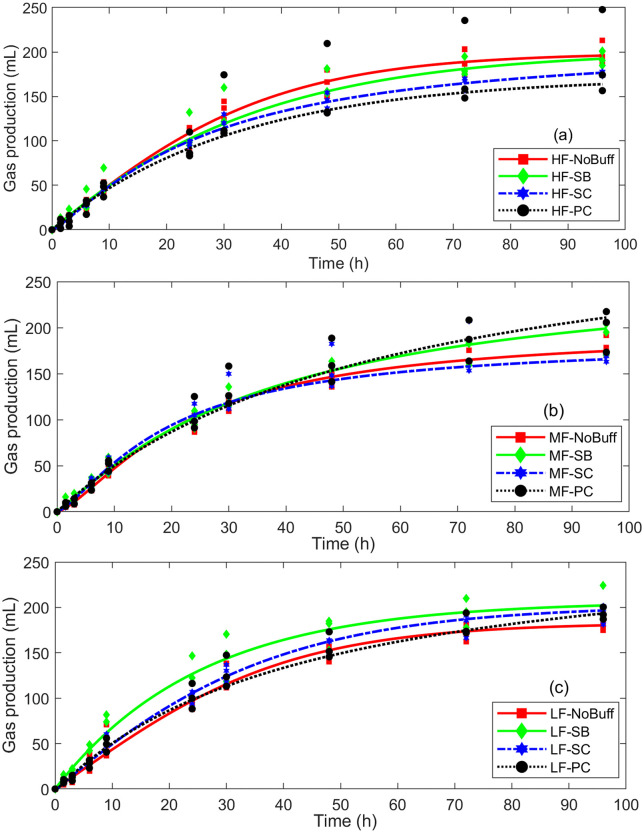
Estimated cumulative response of the Michaelis–Menten model for diets without an acidosis challenge under four buffering treatments over a 96-hour fermentation period. **(a)** high-forage, **(b)** medium-forage, **(c)** low-forage (No Buffer: C-NoBuff; Sodium Bicarbonate: C-SB; Sodium Carbonate: C-SC; Potassium Carbonate: C-PC).

As shown in Fig 2a, all treatments under the high-forage diet without an acidosis challenge exhibited a sigmoidal cumulative response over the 96-hour fermentation period, with clear differences in rate and extent. The unbuffered control (C-NoBuff) showed the steepest initial slope (0–24 hours) and reached the highest final value (195.89), indicating the most rapid and extensive fermentation. Sodium bicarbonate (C-SB) followed closely, with a slightly slower increase and a final value of 192.42. Sodium carbonate (C-SC) and potassium carbonate (C-PC) had flatter curves and lower final values (176.67 and 163.79, respectively), suggesting slower fermentation. Overall, the cumulative response ranked as C-NoBuff > C-SB > C-SC > C-PC, indicating that, under non-acidotic conditions, bicarbonate and no-buffer treatments supported more active fermentation than carbonate-based buffers.

Fig 2b shows the medium-forage diet without an acidosis challenge. All treatments followed a sigmoidal cumulative pattern, but the steepness and final values varied. Potassium carbonate (C-PC) had the fastest initial increase during the first 24 hours and reached the highest final cumulative value (210.97). Sodium bicarbonate (C-SB) followed with 199.04, and sodium carbonate (C-SC) reached 165.63. The unbuffered control (C-NoBuff) had the slowest progression and lowest final value (174.57). The ranking of final values was C-PC > C-SB > C-NoBuff > C-SC, showing that carbonate and bicarbonate buffers, particularly potassium carbonate, enhanced fermentation activity relative to the no-buffer treatment. These findings highlight the buffer-specific and forage-dependent nature of fermentation dynamics.

Under low-forage conditions without acidosis (Fig 2c), sodium bicarbonate (C-SB) produced the fastest and most pronounced response, with a steep increase reaching approximately 78 units by 12 hours and a final cumulative value of 202.15 at 96 hours. Sodium carbonate (C-SC) and potassium carbonate (C-PC) showed moderate responses, reaching 196.61 and 193.37, respectively, while the unbuffered control reached only 180.26. These results indicate that exogenous buffers enhance the system’s fermentation capacity even in the absence of acidotic stress, with sodium bicarbonate showing superior efficacy in both rate and extent, likely due to its higher solubility and immediate availability of bicarbonate ions

**Fig 3:**
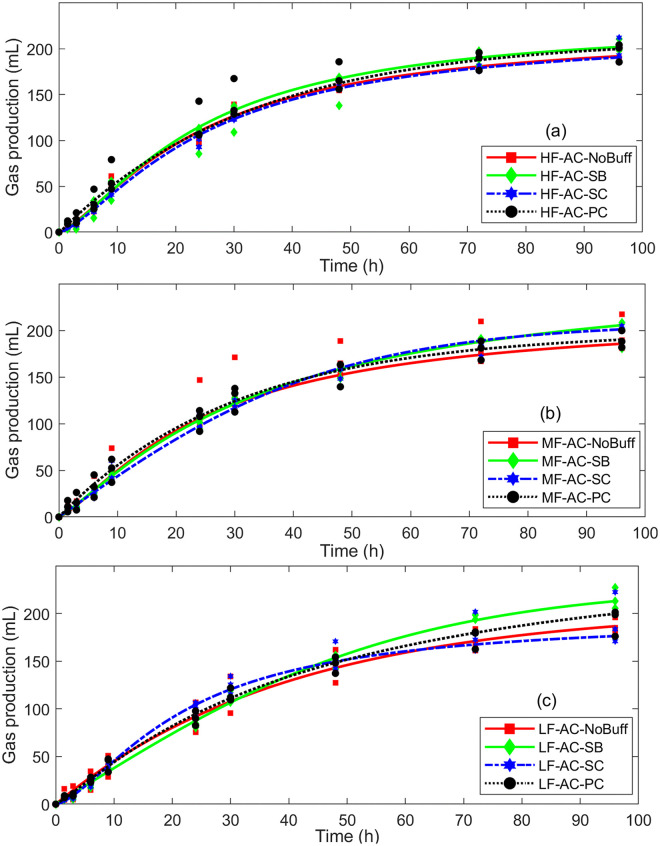
Estimated cumulative responses predicted by the Michaelis–Menten model for diets under an acidosis challenge over a 96-hour fermentation period. Panels show **(a)** high-forage, **(b)** medium-forage, and **(c)** low-forage diets. Treatments include No Buffer (LF_AC-NoBuff), Sodium Bicarbonate (LF_AC-SB), Sodium Carbonate (LF_AC-SC), and Potassium Carbonate (LF_AC-PC).

Fig 3a shows the 96-hour cumulative response of the high-forage diet under an acidosis challenge for four treatments: no buffer, sodium bicarbonate, sodium carbonate, and potassium carbonate. All buffers increased cumulative response compared to the unbuffered control, but with distinct kinetics. Potassium carbonate showed the fastest early-phase increase, rising from 0 at hour 0 to 54.76 units by 10 hours, and reached 199.81 units at 96 hours. Sodium bicarbonate followed with a slightly slower early increase but achieved the highest final value of 201.94 units. Sodium carbonate had a moderate response, reaching 190.53 units at 96 hours, similar to the control (191.92 units) in the late phase despite a slower initial rise. The control consistently had the lowest cumulative response, indicating limited buffering under acidosis. These results indicate that sodium bicarbonate provides the strongest long-term buffering, potassium carbonate offers rapid early mitigation, and sodium carbonate gives moderate improvement over the unbuffered condition.

Fig 3b presents the 96-hour cumulative response for the medium-forage diet under acidosis challenge. All treatments showed rapid early increases due to the moderate fiber content. Potassium carbonate had the steepest early slope (0–10 hours: 0 → 55.44 units) and reached 190.04 units at hour 96. Sodium bicarbonate followed with a slightly slower early rise but the highest final response of 205.60 units, indicating superior long-term buffering. Sodium carbonate reached an intermediate final value of 201.14 units, while the no-buffer control plateaued earlier at 185.72 units, reflecting limited buffering under acidosis. Overall, sodium bicarbonate provided the greatest cumulative benefit, potassium carbonate ensured rapid early-phase mitigation, and sodium carbonate offered moderate improvement.

Fig 3c depicts the 96-hour cumulative response for the low-forage diet under acidosis challenge. All treatments increased rapidly during the initial 0–10 hours, reflecting high fermentability. Potassium carbonate showed the steepest early slope, rising to 44.06 units by 10 hours and reaching 199.77 units at 96 hours, indicating strong early buffering. Sodium bicarbonate had a slightly slower early increase but achieved the highest final response of 212.97 units, demonstrating the greatest long-term buffering capacity. Sodium carbonate reached 176.33 units, while the no-buffer control plateaued at 186.65 units, showing limited fermentation without buffers. All treatments showed slower increases during the late phase (60–96 hours), indicating an approach toward saturation. Overall, sodium bicarbonate provided the most effective cumulative buffering, potassium carbonate offered fast early mitigation, and sodium carbonate gave moderate improvement over the control.

### Rate of gas production under dietary buffers and forage levels

The rate of gas production, calculated as the first derivative of the cumulative gas curve, provides key insights into the dynamic behavior of ruminal fermentation. Unlike cumulative gas volume, which reflects the overall extent of fermentation, the instantaneous rate captures temporal changes in microbial activity and substrate degradation efficiency. Assessing how gas production responds to different buffering strategies and forage levels under both acidotic and non-acidotic conditions offers a detailed understanding of fermentation kinetics. This information is crucial for identifying diet compositions and buffering agents that promote rapid and sustained fermentation in ruminants.

**Fig 4:**
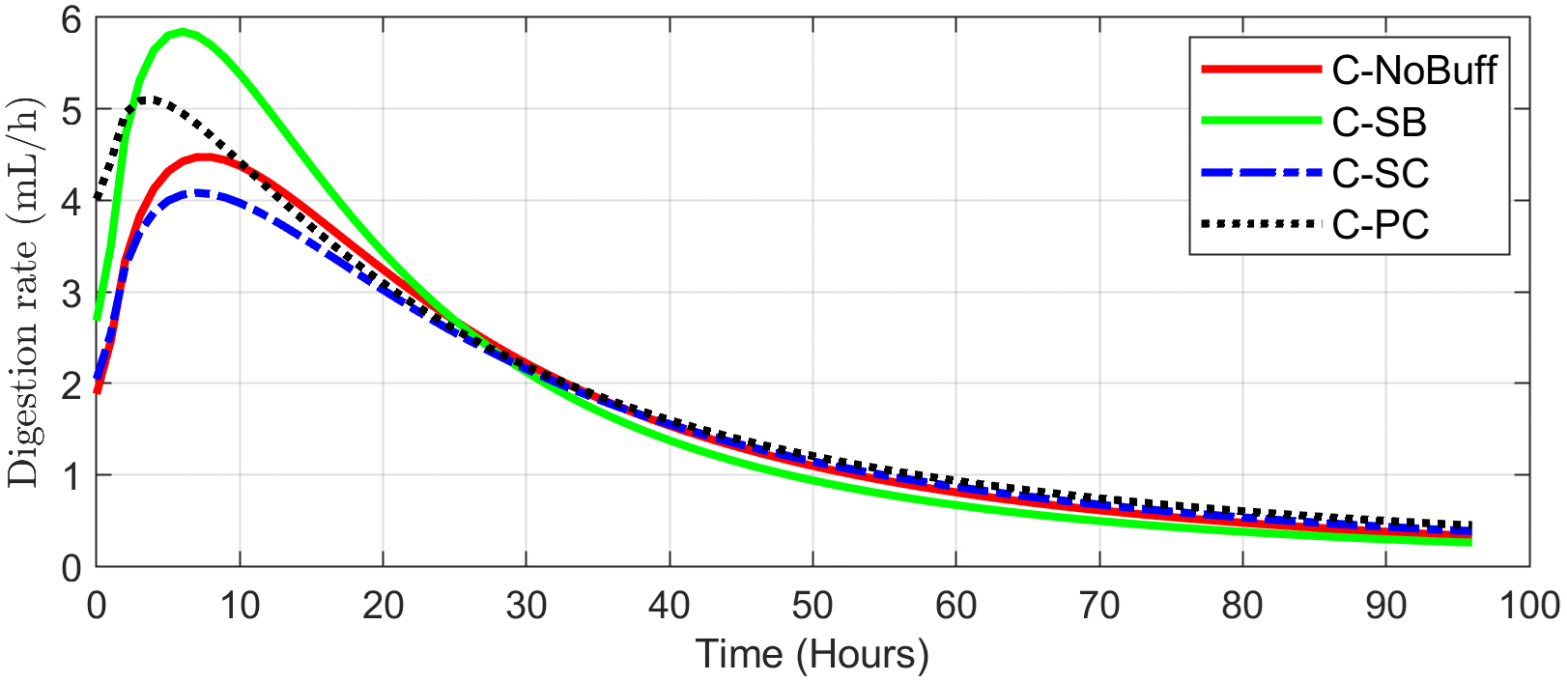
Instantaneous gas production rate during a 96-hour acidosis challenge for the control diet under four buffering treatments. No Buffer (C-NoBuff), Sodium Bicarbonate (C-SB), Sodium Carbonate (C-SC), and Potassium Carbonate (C-PC).

During the 96-hour acidosis challenge, the instantaneous gas production rate (GPR) varied considerably among treatments (Fig 4). Potassium carbonate (C-PC) showed the highest initial GPR, peaking at 5.10 mL/hours at 4 hours and remaining above 4.5 mL/hours until around 9 hours, indicating rapid early fermentation likely due to effective pH stabilization and enhanced microbial activity. Sodium bicarbonate (C-SB) reached a slightly higher peak of 5.84 mL/hours at 6 hours but declined more sharply afterward. Sodium carbonate (C-SC) achieved a lower peak (4.08 mL/hours at 7 hours) yet exhibited a more gradual decrease, reflecting sustained but less intense fermentation. In contrast, the No Buffer treatment (C-NoBuff) showed the lowest GPR, peaking at 4.47 mL/hours around 7–8 hours and declining rapidly thereafter. By 24 hours, GPR values were 2.79 mL/hours for C-NoBuff, compared to 2.68, 2.82, and 2.64 mL/hours for C-PC, C-SB, and C-SC, respectively, highlighting the reduced buffering capacity and likely microbial inhibition under acidotic stress.

After the peak phase, GPR declined gradually for all treatments; however, buffered diets consistently maintained higher rates than the unbuffered control throughout the fermentation period. Among the buffers, C-PC retained the highest GPR at most time points, emphasizing its potential as an effective agent for mitigating acidotic effects and sustaining ruminal fermentation.

### Instantaneous gas production rates across buffers and forage levels

**Fig 5:**
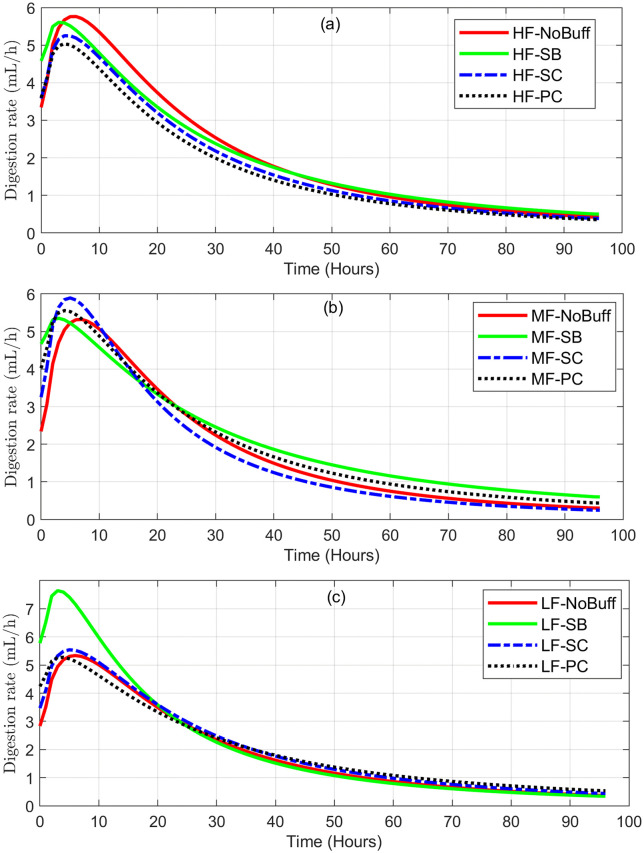
Instantaneous gas production rates during 96-hour fermentations without an acidosis challenge for diets with different forage levels under four buffering treatments. **(a)** high-forage, **(b)** medium-forage, and **(c)** low-forage. Treatments include No Buffer (C-NoBuff), Sodium Bicarbonate (C-SB), Sodium Carbonate (C-SC), and Potassium Carbonate (C-PC).

Fig 5 illustrates the temporal dynamics of instantaneous gas production rates (GPR) over the 96-hours incubation period, highlighting the effects of different buffering agents across varying forage levels.

Under the high-forage diet (Fig 5a), all treatments showed a clear peak in GPR within the first 6 hours. Sodium bicarbonate (C-SB) consistently produced the highest rates (e.g., 5.50 mL/hours at 2 hours), reflecting its strong buffering capacity and early enhancement of microbial activity. After the peak, GPR gradually declined in all treatments, with C-SB maintaining slightly higher rates than the other buffers up to 24 hours. The no-buffer control (C-NoBuff) showed the steepest post-peak decline, suggesting greater acid accumulation and inhibition of fermentation. Potassium carbonate (C-PC) exhibited the lowest rates during mid- to late fermentation, possibly due to lower buffering efficacy or altered microbial efficiency.

In the medium-forage diet (Fig 5b), early-phase GPR was generally lower than in the high-forage treatment, particularly for C-NoBuff. Sodium bicarbonate again showed early superiority (5.35 mL/hours at 3 hours), but sodium carbonate (C-SC) surpassed it between 3 and 6 hours, peaking at 5.89 mL/hours at 5 hours. This indicates a potential interaction between forage level and buffer type affecting fermentation kinetics. The subsequent decline in GPR resembled that of the high-forage treatment but was more pronounced in SC and PC treatments, likely due to lower availability of fermentable carbohydrates in the moderately fibrous substrate.

For the low-forage diet (Fig 5c), differences among buffering strategies were more pronounced. Sodium bicarbonate promoted the fastest early increase, reaching the maximum GPR observed across all treatments (7.64 mL/hours at 3 hours), reflecting the high fermentability of the starch-rich substrate. However, this was followed by a sharp decline, likely due to rapid substrate depletion or microbial feedback inhibition. Both C-NoBuff and C-PC showed lower and similar trajectories, while C-SC maintained intermediate and more stable GPR throughout the period, indicating sustained fermentation activity.

Overall, these results highlight the critical role of buffers in regulating fermentation dynamics. Sodium bicarbonate provided rapid and robust effects across all forage levels, sodium carbonate offered more prolonged stabilization, especially in low-forage diets, and potassium carbonate showed limited efficacy. The consistently lower performance of C-NoBuff emphasizes the importance of extracellular pH regulation in supporting efficient ruminal fermentation, even in the absence of an induced acidosis challenge.

### Instantaneous gas production during acidosis challenge across forage levels and buffers

**Fig 6:**
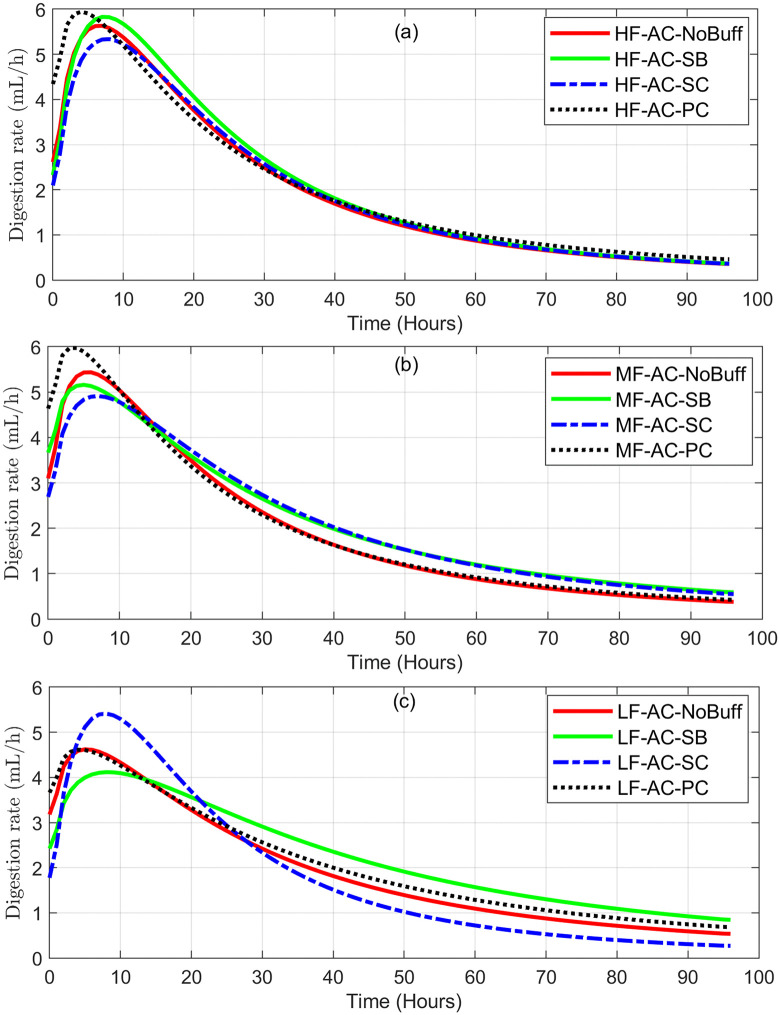
Instantaneous gas production rates during a 96-hour acidosis challenge for diets with varying forage levels under four buffering treatments. **(a)** high-forage, **(b)** medium-forage, and **(c)** low-forage. Treatments include No Buffer (LF_AC-NoBuff), Sodium Bicarbonate (LF_AC-SB), Sodium Carbonate (LF_AC-SC), and Potassium Carbonate (LF_AC-PC).

Fig 6 shows the dynamics of instantaneous gas production (GPR) over a 96-hour acidosis challenge for high-, medium-, and low-forage diets under four buffering treatments: no buffer, sodium bicarbonate (SB), sodium carbonate (SC), and potassium carbonate (PC).

For the high-forage diet (Fig 6a), all treatments peaked around hour 6. Potassium carbonate (PC) achieved the highest GPR (5.94 mL/g DM), followed by SB and SC. The no-buffer group exhibited consistently lower rates and a more gradual decline, likely due to delayed acid accumulation associated with the higher structural fiber content. PC maintained elevated gas production longer than other buffers, reflecting its strong buffering capacity and resistance to ruminal pH drops.

In the medium-forage diet (Fig 6b), treatment differences were more pronounced. SB and PC produced the highest initial gas rates, peaking between 4 and 6 hours (5.97 mL/g DM for both), while the no-buffer group plateaued at a lower value (~5.43 mL/g DM). After 12 hours, the no-buffer group declined more sharply, whereas buffered treatments maintained higher GPR, indicating stabilization of fermentation. PC showed superior performance in the post-peak phase, suggesting improved microbial resilience.

For the low-forage diet (Fig 6c), which is more prone to rapid acidotic shifts due to high fermentable carbohydrate content and low effective fiber, treatment differences were most evident. Peak GPR occurred earlier (4–6 hours), with SB reaching 5.41 mL/g DM. Sodium carbonate (SC) maintained higher mid-phase gas production (above 5.0 mL/g DM from 5–12 hours), outperforming other treatments during this period. The no-buffer group declined rapidly post-peak, falling below 4.0 mL/g DM by 11 hours, reflecting accelerated acidotic stress and reduced microbial activity.

Across all forage levels, buffers enhanced gas production rates and prolonged fermentation under acidotic conditions. PC consistently performed best in high- and medium-forage diets by sustaining elevated GPR over an extended period. In contrast, SC was most effective in low-forage diets, counteracting rapid pH drops and supporting sustained microbial activity. These findings emphasize that both forage level and buffer selection are critical for managing ruminal fermentation under acidosis. Buffer efficacy is forage-dependent: PC shows broad effectiveness, while SC is particularly beneficial in rapidly fermentable, low-forage diets due to its carbonate-based pH stabilization and ion-exchange properties.

Figs 4–6 summarize the maximum instantaneous gas production rates (Max GPR) and the times at which they occurred (Time at Max) across different diets, buffering agents, and experimental conditions (control vs. acidosis challenge).

Under control conditions without acidosis, sodium bicarbonate (C-SB) produced the highest Max GPR (5.84 mL/hours at 6 hours), followed by potassium carbonate (C-PC, 5.10 mL/hours at 4 hours). The no-buffer treatment (C-NoBuff) reached a lower peak (4.47 mL/hours at 7 hours), indicating limited fermentation efficiency without pH stabilization.

The response also depended on the forage level. In high-forage diets without acidosis, C-NoBuff achieved the highest Max GPR (5.76 mL/hours at 5–6 hours), likely due to the natural buffering effect of structural fiber. Sodium bicarbonate and sodium carbonate also reached high rates (5.61 mL/hours and 5.25 mL/hours, respectively), but at earlier times, reflecting faster microbial activity. In medium-forage, non-acidotic diets, sodium carbonate (5.89 mL/hours at 5 hours) outperformed other treatments, suggesting a favorable interaction between moderate fiber content and SC’s buffering properties. In low-forage diets, which are highly fermentable, sodium bicarbonate markedly enhanced Max GPR (7.64 mL/hours at 3 hours), demonstrating rapid buffering and effective fermentation in starch-rich conditions.

Under the acidosis challenge, Max GPR patterns shifted. In high-forage diets, potassium carbonate achieved the highest peak (5.94 mL/hours at 4 hours), highlighting its ability to sustain early microbial activity under acidotic stress. Sodium bicarbonate and sodium carbonate reached their maxima later (7–8 hours), indicating delayed but sustained buffering. In medium-forage acidotic diets, PC again showed the highest Max GPR (5.97 mL/hours at 3–4 hours), followed by C-SB (5.16 mL/hours at 5 hours), confirming its consistent efficacy. In low-forage acidotic conditions, sodium carbonate provided the highest peak (5.41 mL/hours at 8 hours), whereas PC and SB peaked earlier but at lower values, likely reflecting rapid microbial inhibition due to low fiber and intensified acid stress.

Overall, these results highlight the complex interplay between forage level, buffer type, and acidosis on fermentation kinetics. Sodium bicarbonate was most effective for enhancing early gas production, especially in low-forage, non-acidotic diets. Potassium carbonate consistently supported high fermentation rates under acidosis, particularly in high- and medium-forage diets. Sodium carbonate provided stable buffering in low-forage, acidotic diets, likely due to slower ion release and sustained pH stabilization. These findings emphasize the importance of tailoring buffering strategies to diet composition and physiological conditions to optimize rumen fermentation.

## Conclusion

In this study, 32 candidate nonlinear models were initially evaluated to describe ruminal fermentation kinetics across diverse dietary compositions and buffering treatments. From these, the 10 top-performing models were shortlisted based on a composite performance score incorporating goodness-of-fit metrics and biological plausibility. Among the shortlisted models, the Michaelis–Menten model was selected for detailed analysis because all its parameters were consistently statistically significant across dietary treatments, and the model offered interpretability and simplicity. This selection ensured a parsimonious framework capable of accurately capturing overall fermentation kinetics.

The Michaelis–Menten model exhibited high predictive accuracy (R² and adjusted R² ≈ 0.97–1.00) and low-to-moderate RMSE values, effectively representing cumulative gas production under both normal and acidotic conditions. Parameter analysis indicated that maximum fermentation capacity (b_1_), substrate affinity (b_2_), and basal microbial activity (b_3_) were strongly affected by forage level and buffer type. Sodium bicarbonate (SB) and potassium carbonate (PC) enhanced fermentation potential, improved substrate utilization, and sustained basal microbial activity, particularly under acidotic conditions, whereas sodium carbonate (SC) provided moderate but stable buffering, underscoring the treatment-specific effects on fermentation dynamics.

Cumulative and instantaneous gas production analyses revealed treatment-specific patterns: SB accelerated early-phase fermentation in low-forage diets, PC supported prolonged fermentation in medium- and high-forage diets under acidotic stress, and SC maintained steady activity in rapidly fermentable, low-forage diets. These results highlight the critical role of both forage composition and buffer selection in optimizing rumen fermentation, mitigating subacute acidosis, and stabilizing microbial activity.

The Michaelis–Menten model exhibited high statistical robustness, clear biological interpretability, and practical simplicity in characterizing ruminal fermentation kinetics. Experiments were conducted under controlled in vitro conditions; nevertheless, the results offer valuable guidance for optimizing dietary formulations and buffer application strategies. Future research should validate these findings *in vivo*, examine a wider range of feed compositions, and assess the long-term effects of different buffering strategies on rumen function and overall animal performance.
